# Using the Steady-State Chloride Migration Test to Evaluate the Self-Healing Capacity of Cracked Mortars Containing Crystalline, Expansive, and Swelling Admixtures

**DOI:** 10.3390/ma12111865

**Published:** 2019-06-09

**Authors:** Fahad ul Rehman Abro, Abdul Salam Buller, Kwang-Myong Lee, Seung Yup Jang

**Affiliations:** 1 Department of Civil, Architectural, and Environmental Systems Engineering, Sungkyunkwan University (SKKU) 2066, Seobu-ro, Jangan-gu, Suwon, Gyeonggi-do 16419, Korea; fahad.abro@skku.edu (F.u.R.A.); abdulsalam@skku.edu (A.S.B.); leekm79@skku.edu (K.-M.L.); 2 Graduate School of Transportation, Korea National University of Transportation, 157, Chuldo-bangmulgwan-ro, Uiwang, Gyeonggi-do 16106, Korea

**Keywords:** chloride, concrete, crack, mortar, self-healing, steady-state migration

## Abstract

Interest in self-healing-crack technologies for cement-based materials has been growing, but research into such materials remains in the early stage of development and standardized methods for evaluating healing capacity have not yet been established. Therefore, this study proposes a test method to evaluate the self-healing capacity of cement-based materials in terms of their resistance to chloride penetration. For this purpose, the steady-state chloride migration test has been used to measure the diffusion coefficients of cracked mortar specimens containing crystalline, expansive, and swelling admixtures. The results of the present study show that the time to reach a quasi-steady-state decreased and the diffusion coefficients increased as the potential increased because of the potential drop inside the migration cell and self-healing that occurred during the test. Therefore, use of a high potential is recommended to minimize the test duration, as long as the temperature does not rise too much during the test. Using this test method, the self-healing capacity of the new self-healing technologies can be evaluated, and an index of self-healing capacity is proposed based on the rate of charged chloride ions passing through a crack.

## 1. Introduction

Concrete is the most widely used construction material because it is economical and easy to use in buildings. However, cracks develop in most concrete structures through a variety of causes, such as shrinkage due to moisture and temperature variation, expansive chemical reactions, and mechanical loading. The durability of concrete structures can be unfavorably affected by cracks, which provide an easy way for water, chloride ions, or gases to penetrate into the concrete, thus increasing the transport properties of the concrete [1]. In other words, cracks can rapidly increase the rate of chloride ingress and carbonation, which are the two main causes of reinforcement corrosion and the subsequent significant deterioration of concrete structures [2]. To prevent the deterioration of structures in practice, immediate repairs are required upon the formation of cracks to prevent the ingress of moisture, chloride, and carbon dioxide gas. However, crack repair is both costly and labor-intensive [3]. 

Therefore, interest in self-healing-crack technology is growing. Self-healing cracks can minimize the ingress of chloride ions and other aggressive matter without the need for manual crack repairs. Autogenous healing was already observed by the French Academy of Science in 1836 [4], and several studies during the last century have unveiled the presence of the autogenous self-healing capability of cementitious composites due to delayed hydration of binder and/or carbonation [5,6]. Recently, various techniques to enhance the autogenous healing of concrete by incorporating mineral additives or crystalline admixtures are under development [7,8,9]. Some autonomous healing technologies that use super-absorbent polymers, encapsulated polymers, minerals, or bacteria are also being studied [10,11,12,13].

For self-healing technology to be adopted in practical use, a unified and standardized way to evaluate the self-healing capacities of different techniques is required. So far, the bending test, permeability test, non-destructive tests, or microscopic examination have been used to evaluate the capacity for self-healing [10,14,15,16]. However, a limited amount of research has considered the evaluation of self-healing capacity with regard to chloride penetration resistance [10,12,17,18]. Since the self-healing capacity chloride penetration can only indirectly reflect the self-healing capacity, the self-healing capacity with respect to chloride penetration resistance should be assessed with a quantitative parameter so that it can be correlated and calibrated with actual amount of crack healing.

Resistance to chloride penetration has been intensely studied around the world since the late 1970s. In particular, since the beginning of the 1980s, many experimental methods have been proposed to estimate the diffusivity of chloride ions in concrete. Several test methods, including the immersion or ponding test [19,20,21,22,23,24,25,26,27], have been adopted as standard test methods. Since the late 1990s, several studies have examined the chloride penetration of cracked concrete using different test methods [28,29,30,31,32,33,34,35,36,37,38,39,40].

In the immersion or ponding test, the specimen, pre-saturated with water and fully coated with sealant except for one exposure surface, is immersed in or ponded with chloride solution for a specified duration. This test can simulate the natural mechanism of chloride penetration into cement-based materials. However, the test duration of the immersion test is, in general, 90 days to 6 months, which is too long to determine a change in resistance to chloride penetration due to self-healing. Also, it is a labor-intensive process, requiring the collection of dust samples at several different depths to measure their chloride content.

Therefore, many researchers have applied an external electrical field to accelerate the test by enhancing chloride transport for investigating the resistance of cracked concrete to chloride penetration. Şahmaran et al. [33] used the test method in ASTM C1202 [25] to examine chloride penetration in cracked and uncracked cementitious composite specimens with crack widths of 50 to 140 µm, assessing the self-healing capacity 60 days after cracking. The ASTM C1202 test method, known as “the Coulomb test,” uses the total charge passed during the first 6 h of the test while applying a 60 V potential as an index for the resistance to chloride ion penetration. Although it takes a very short time to determine the results, this method cannot give information about chloride diffusion specifically because the charge passed is related to all ions transported through concrete, not just chloride ions, and therefore, no theoretical relationship between the charge passed and the chloride diffusion coefficients can be established [41]. Using the same test setup, however, the steady-state diffusion coefficient can be obtained through a theoretical calculation [41] adopted in NT Build 355 [26]. Jacobsen et al. [28] applied this approach to determine the steady-state chloride diffusion coefficients of cracked specimens to assess their self-healing capability under different curing conditions. They observed an increase in the chloride diffusion coefficient due to the crack, and 28%–35% reduction after healing. Darquennes et al. [17] studied the self-healing of cracked concrete incorporating blast-furnace slag at early age using the steady-state chloride migration test, and they observed a linear relationship between crack width and diffusion coefficient. Şahmaran [18] also studied the diffusion coefficients of cracked specimens with various sizes of crack widths 29–390 µm, and found that for a crack width ≤135 µm, the effect on diffusion coefficient was marginal whereas for the crack width lager than 135 µm, a rapid increment in the diffusion coefficient was observed. Furthermore, for the cracks lower than 50 µm, a substantial healing was noticed. Jang et al. [1] applied a similar method to test the steady-state diffusion coefficients of cracked concrete specimens and also found a linear relationship between crack width and the chloride diffusion coefficient above a certain critical crack width. However, that steady-state migration test takes 1–2 weeks for the steady-state to be reached, even in normal-strength concrete, making it too long to evaluate self-healing capacity because the specimens are always in contact with water (i.e., chloride solution), and thus, self-healing could occur during the test [17,42].

In this regard, this study proposes an appropriate test method to evaluate the self-healing capacity of cracked cementitious materials with respect to their resistance to chloride penetration. For this purpose, the electrically driven rapid migration test was used to determine the diffusion coefficients of cracked mortar in a relatively short time. The test procedure is presented, including specimen preparation and the steady-state migration test. The effect of the applied potential was investigated, and the test procedure was applied to evaluate the self-healing capacity of cracked mortars containing crystalline admixtures with or without an expansive admixture and swelling agent. Finally, an index for self-healing capacity in terms of resistance to chloride penetration is proposed.

## 2. Theoretical Background

Two different methods can determine the diffusion coefficient of concrete through the electrical migration test: One calculates it under the non-steady-state regime and the other under the steady-state regime. 

Tang and Nilsson [43] developed a method to determine the diffusion coefficient from the chloride penetration depth measured by the colorimetric method using silver nitrate and based on an equation derived under the non-steady state regime; their method has been adopted as a Norwegian test standard—NT Build 492 [27]. However, chloride transport through a crack is too rapid to determine the chloride penetration depth in the crack, and thus, it is difficult to apply this method to cracked concrete.

Instead, it is more convenient to apply the steady-state migration test to cracked concrete because the chloride flux is constant under the steady-state and is divided into fluxes through the cracked and uncracked parts, proportional to their areas. 

In the migration test cell, the chloride flux is expressed by the Nernst–Planck equation [44] as follows.

(1) $$J_{c}=\frac{zF}{RT}Dc\frac{\partial U}{\partial x}$$

Here, $J_{c}$ is the chloride flux, z is the ionic valence, F is the Faraday constant (=96,485 C per equivalent), R is the gas constant (=8.3145 J/mol·K), T is the absolute temperature [K], D is the diffusion coefficient, c is the chloride concentration, U is the electrical potential applied [V], and x is the distance. Therefore, the diffusion coefficient under the steady-state condition is determined by the following.

(2) $$D_{ssm}=\frac{RTL}{zFU}\frac{J_{c}}{c_{1}}$$

Here, $D_{ssm}$ is the diffusion coefficient [m^2^/s] obtained from the steady-state migration test, L is the specimen thickness [m], and $c_{1}$ is the chloride concentration in the upstream cell (catholyte). The chloride flux under the steady-state condition can be measured during the test by the following equation.

(3) $$J_{c}=\frac{V}{A}\left|\frac{\Delta c_{1}}{\Delta t}\right|=\frac{V}{A}\left|\frac{\Delta c_{2}}{\Delta t}\right|$$

Here, A is the cross-sectional area [m^2^] through which ions pass, V is the volume of the upstream or downstream cell [m^3^], and $c_{2}$ is the chloride concentration in the downstream cell (anolyte). According to Jang et al. [1], under the steady-state condition, the rate of concentration drop in the upstream cell is almost same as the rate of increase in the downstream cell. Therefore, the diffusion coefficient can be determined from the rate of concentration drop in the upstream cell as follows.

(4) $$D_{ssm}=\frac{RTL}{zFU}\frac{V}{A}\frac{1}{c_{1}}\left|\frac{\Delta c_{1}}{\Delta t}\right|=\frac{RTL}{zFU}\frac{V}{A}\left|\frac{\Delta \ln\left(c_{1}\right)}{\Delta t}\right|$$

Because the chloride concentration in the upstream cell, $c_{1}$, also changes during the test, it is herein referred to as a “quasi-steady-state” when $\Delta \ln\left(c_{1}\right)/\Delta t$ becomes constant, following Page et al.’s [45] definition [1].

## 3. Experimental Program

### 3.1. Test Outline

The experimental program is divided into two phases. In the first phase, preliminary tests were conducted to verify whether the method is suitable for evaluating the self-healing performance of cracked specimens and to determine the relevant electrical potential. For this purpose, three different potentials (12 V, 24 V, and 36 V) were applied to cracked and uncracked ordinary Portland cement (OPC) specimens with 0.1 and 0.3 mm crack widths immediately after the preparation of the cracked specimens. For each applied potential, the tests were performed three times on the same specimen to check the repeatability of the test. In the second phase, the self-healing capacities of cracked specimens incorporating different admixtures were evaluated. The major variables in this phase were the types of admixtures and the crack widths, which ranged from 0 to 0.5 mm. The tests were performed on cracked and uncracked specimens before and after healing (of 28 and 56 days). Before the healing of cracks, the tests were repeated with varying crack widths, as shown in Table 1, to study the relationship between the crack width and the diffusion coefficient of the same specimen. 

### 3.2. Materials and Mix Proportions

The mixture proportions of the test mortar specimens are summarized in Table 2. Type I ordinary Portland cement and two different types of self-healing materials were used as binders. The SH-A mixture incorporated a crystalline admixture of Na_2_CO_3_ and organic Ca^2+^ together with zeolite. The SH-B mixture included calcium sulfoaluminate (CSA) as an expansion admixture and bentonite as a swelling agent together with the crystalline admixture in SH-A. The density of the fine aggregate used was 2.68 g/cm^3^. The slump flow of the mixtures and the compressive strength measured at 28 days are also summarized in Table 2.

### 3.3. Preparation of Cracked Specimens

**Figure 1:**
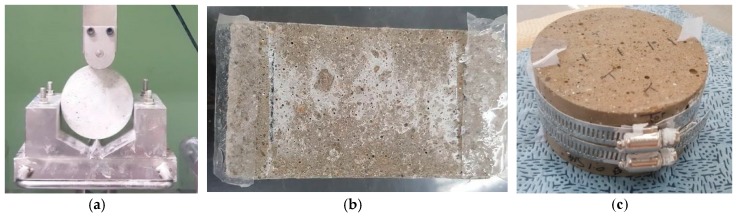
Preparation of cracked specimens: (**a**) splitting; (**b**) adhering silicon tapes; (**c**) reassembling.

Cylindrical specimens of Ø100 mm × 200 mm were manufactured and demolded after 24 h. The central part of each cylindrical specimen was cut into 50 mm disks. After 28-day water curing in a water bath at a constant temperature of 20 ± 2 °C, the specimens were split into two semi-circles under a compressive load, as shown in Figure 1a. After that, the two pieces were reassembled using a steel band, as shown in Figure 1c. The target crack widths were achieved using silicon tapes as thick as the target crack width, adhering the tapes to the ends between the two semi-circular pieces [46], as shown in Figure 1b. 

The actual crack widths were measured at three points on the top and bottom of each specimen using an optical microscope immediately after reassembling of each cracked specimen. The mean value was taken as the crack width of the specimen. The targeted and achieved crack widths in the first phase test specimens are presented in Table 3: The differences between the target and measured crack widths are less than 10%.

The cracked specimens were tested before self-healing, and then, to allow the self-healing process to continue, they were immersed in the water bath at a temperature of 20 ± 2 °C until the next test. The specimens with each mixture proportion were cured separately from the specimens with the other mixture proportions to prevent ion-exchange between them.

### 3.4. Steady-State Chloride Migration Test

**Figure 2:**
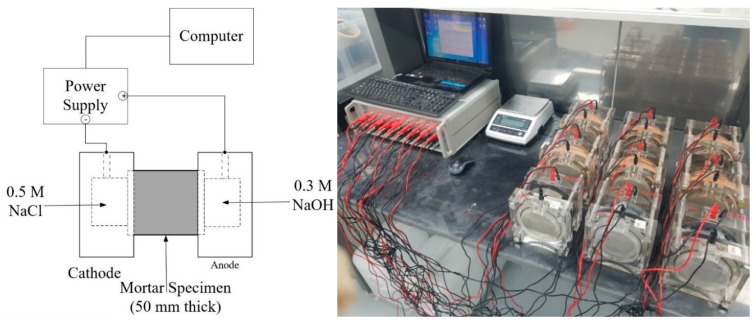
Test set-up for chloride migration testing.

In this study, the test setup of ASTM C1202 was adopted to measure the steady-state diffusion coefficient of the cracked concrete, but the applied potential and test duration were changed from the original test method. As explained in Section 3.1, different potentials (12, 24, and 36 V) were applied in the first-phase tests to examine the effects of the electrical potentials on the test results. In the second-phase test, the potential selected from the first-phase test, 36 V, was applied. The upstream cell (catholyte) was filled with 0.5 M NaCl solution, and the downstream cell (anolyte) was filled with 0.3 M NaOH solution, following ASTM C1202. Each specimen was kept between those two cells with a rubber seal to prevent leakage. Figure 2 presents a schematic diagram and photograph of the test setup.

Before the test, the specimens were pre-conditioned with vacuumed pressure in the desiccator for 12 h to remove air from the pores and allow the specimen to be fully saturated with water. The test was conducted in a room with a controlled temperature of 20 ± 2 °C and relative humidity of 60 ± 5%.

**Figure 3:**
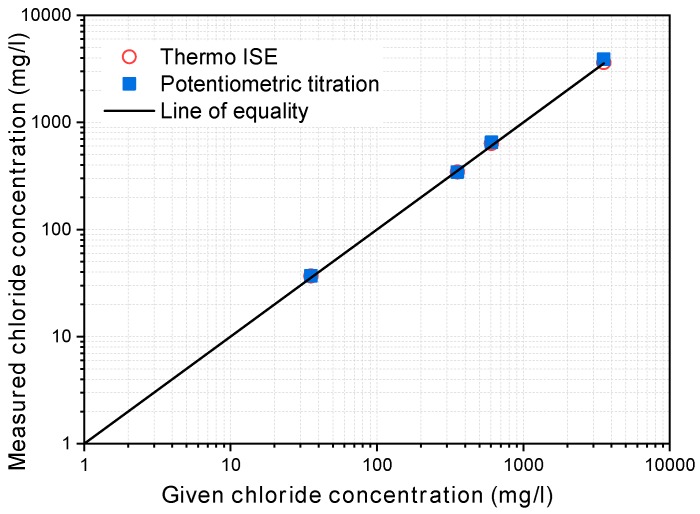
Verification of the chloride concentration measured by the ion selective electrode (ISE).

The chloride ion concentration in the upstream cell was periodically measured with an ion selective electrode (ISE), Thermo Scientific ISE produced by Orion Scientific (Waltham, MA, USA) [47]. Based on the pre-determined relationship between the potential obtained by the ISE and the known chloride concentration, measurements were made once an hour for the first 6 h. After that, the intervals were readjusted according to the rate of change of chloride concentration. At each measurement, a small volume of chloride solution was taken from the upstream cell using a pipette. The results obtained by the ISE were verified by comparing them with the concentrations measured by potentiometric titration, as prescribed in ASTM D512-12 [48], and the given concentrations. As shown in Figure 3, the concentrations measured by both potentiometric titration and the ISE were almost the same and lie on the line of equality, which confirms the accuracy of the chloride concentration measured by the ISE.

## 4. Results and Discussion

### 4.1. Effect of Electrical Potential

**Figure 4:**
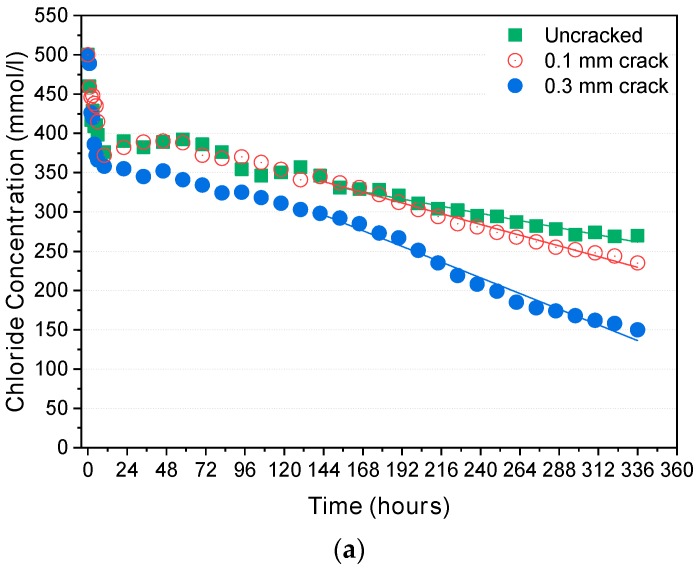

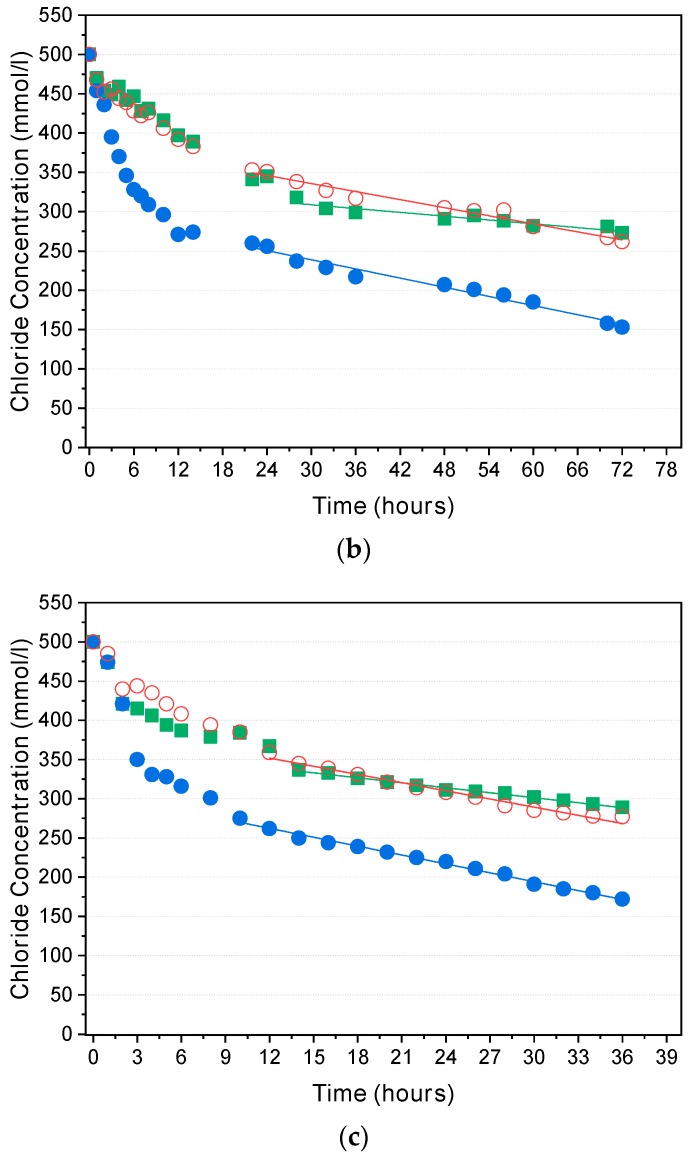
Chloride concentration drops in the upstream cell: (**a**) 12 V; (**b**) 24 V; (**c**) 36 V.

**Figure 5:**
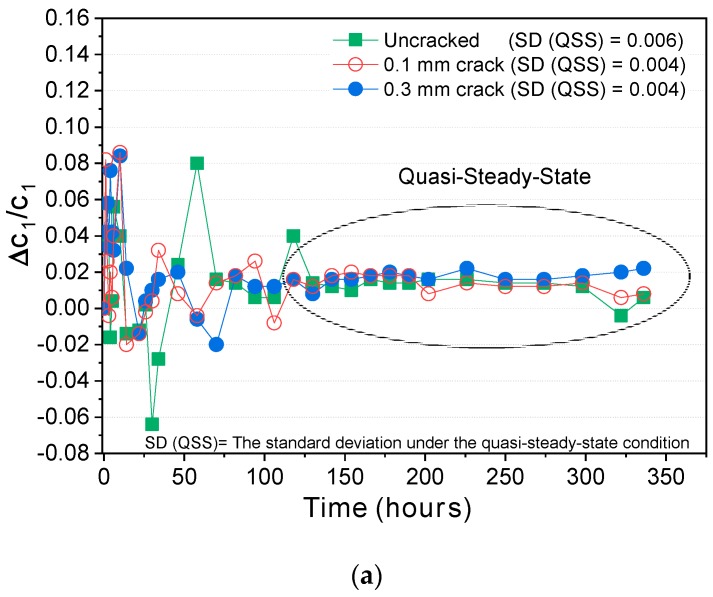

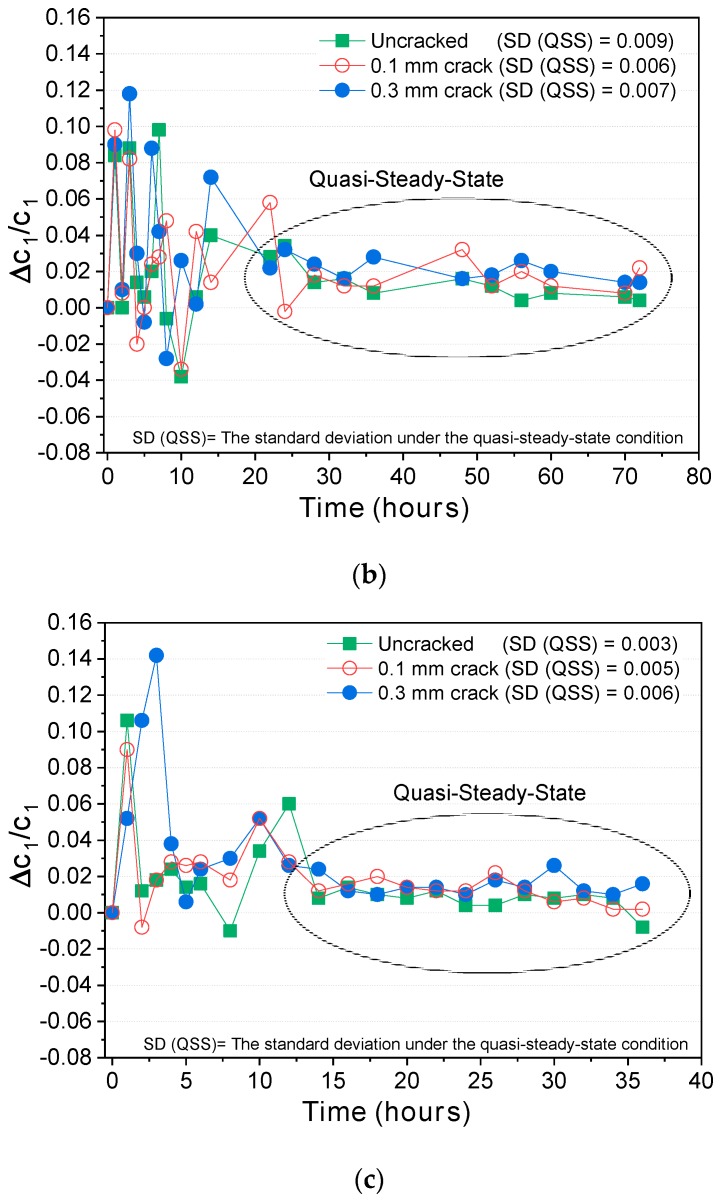
Variation in the rate of chloride concentration drops ($\Delta c_{1}/c_{1}$) over time: (**a**) 12 V; (**b**) 24 V; (**c**) 36 V.

The chloride concentration in the upstream cell for the cracked and uncracked specimens was measured during the test, and the change in chloride concentration over time is depicted in Figure 4. The chloride concentration dropped rapidly, and the rate of concentration drop was not constant in the initial stage. After a certain period, it became almost constant as the quasi-steady-state was reached. Plotting the change in $\Delta c_{1}/c_{1}$ (Figure 5) clearly confirms whether the quasi-steady-state has been reached. The standard deviation of $\Delta c_{1}/c_{1}$ under the quasi-steady-state condition was within 1%.

It is clearly shown in Figure 4 that the slope of the concentration drop becomes higher as the cracks become wider. Figure 5 also shows that the time to reach the quasi-steady-state depends on the potential applied: Under 12 V, it took more than 150 h for the uncracked specimen and 100–120 h for the cracked specimens. Under 24 V and 36 V, the chloride concentration decreased more rapidly, and the quasi-steady-state was reached more quickly than under 12 V: 22–26 h for the cracked and uncracked specimens under 24 V and 12–15 h under 36 V. 

**Figure 6:**
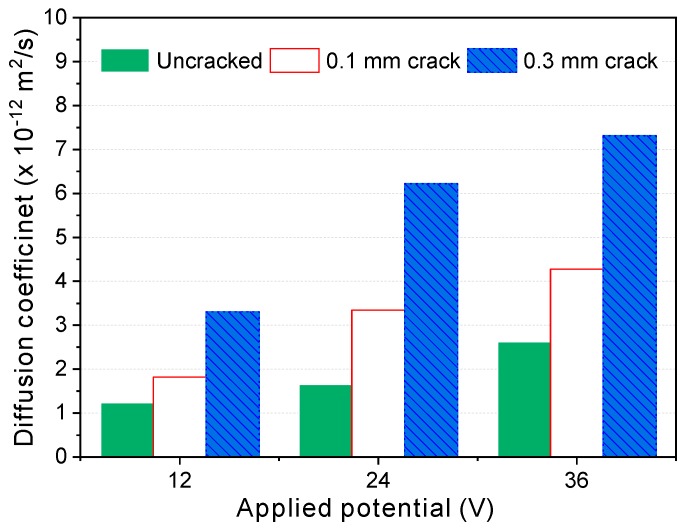
Diffusion coefficients according to the applied potential and crack width.

Figure 6 shows the calculated steady-state migration diffusion coefficients $D_{ssm}$ according to the crack width and applied potential. As can be seen in this figure, the diffusion coefficient increases along with the applied potential and crack width. It is worth mentioning here that the increase in the $D_{ssm}$ of the cracked specimens was larger when the applied potential changed from 12 to 24 V than when it changed from 24 to 36 V, whereas that of the uncracked specimen was smaller when changing from 12 to 24 V than from 24 to 36 V. That result implies that self-healing of the crack might occur during the test, as pointed out by [17]. Under 12 V, the degree of self-healing during the test might be larger because the test duration was almost 14 days (330 h), much longer than the others. According to the test results in [49] and [50], self-healing occurs very early, in less than a week. Undoubtedly, therefore, a short test duration is crucial in evaluating the diffusion coefficient of cracked specimens.

**Figure 7:**
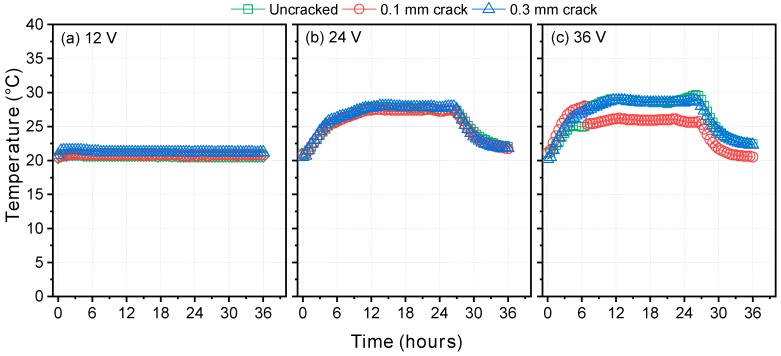
Temperature rise in the chloride solution in the upstream cell during the test: (**a**) 12 V; (**b**) 24 V; (**c**) 36 V.

Other reasons could explain the larger diffusion coefficient under higher potential. One possible reason is the temperature rise caused by high voltage. Figure 7 shows the rise in the temperature in the chloride solution in the upstream cell during the test: From 20 °C at the beginning of the test to 28 °C and 29.5 °C under 24 V and 36 V, respectively, decreasing again after a certain time, whereas the temperature rise under 12 V was only 1.5 °C. Nonetheless, the temperature under 24 and 36 V remained below 40 °C, which is the maximum temperature allowed by NT Build 355 [26], and was less than 4% of 293 K (20 °C).

Another possible reason is the potential drop throughout the cell caused by polarization. According to McGrath and Hooton [51], the potential drop depends slightly on, but is not proportional to, the magnitude of the potential applied, and therefore, the error caused by the potential drop increases as the applied potential decreases. In other words, higher potential is more appropriate for evaluating the effect of a crack on the diffusion coefficient and self-healing capacity unless it induces too high a temperature rise. For these reasons, 36 V was selected for the second-phase testing.

### 4.2. Effect of Crystalline and Expansive Admixtures on the Self-Healing of Cracks

For the second phase of testing, which evaluated the capacity for self-healing of a crack in the mortar, steady-state migration tests under the potential of 36 V were conducted on mortar with and without crystalline and expansive admixtures.

**Figure 8:**
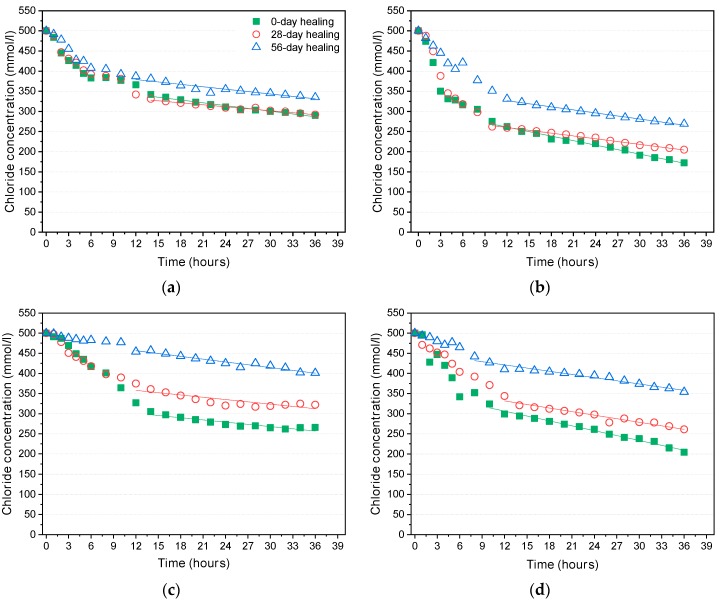

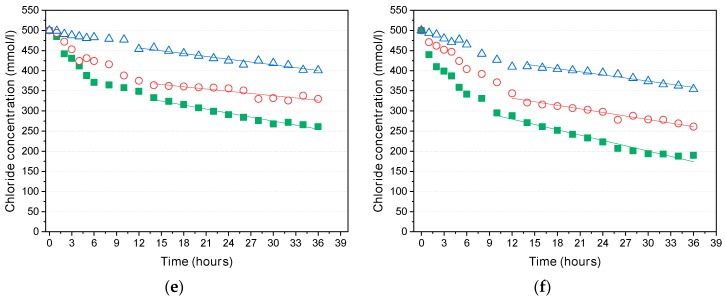
Chloride concentration drop in the upstream cell: (**a**) ordinary Portland cement (OPC), uncracked; (**b**) OPC, 0.3 mm crack; (**c**) SH-A, uncracked; (**d**) SH-A, 0.3 mm crack; (**e**) SH-B, uncracked; (**f**) SH-B, 0.3 mm crack.

The rate of drop in the chloride concentration in the upstream cell during tests of the various mixtures before and after healing is illustrated in Figure 8. As already shown in the first-phase tests, the rate of concentration drop for cracked specimens (0.3 mm crack) was larger than the rate for uncracked specimens. After 28 and 56 days of healing (or additional curing), the rates of the concentration drops were reduced for both the cracked and uncracked specimens.

**Figure 9:**
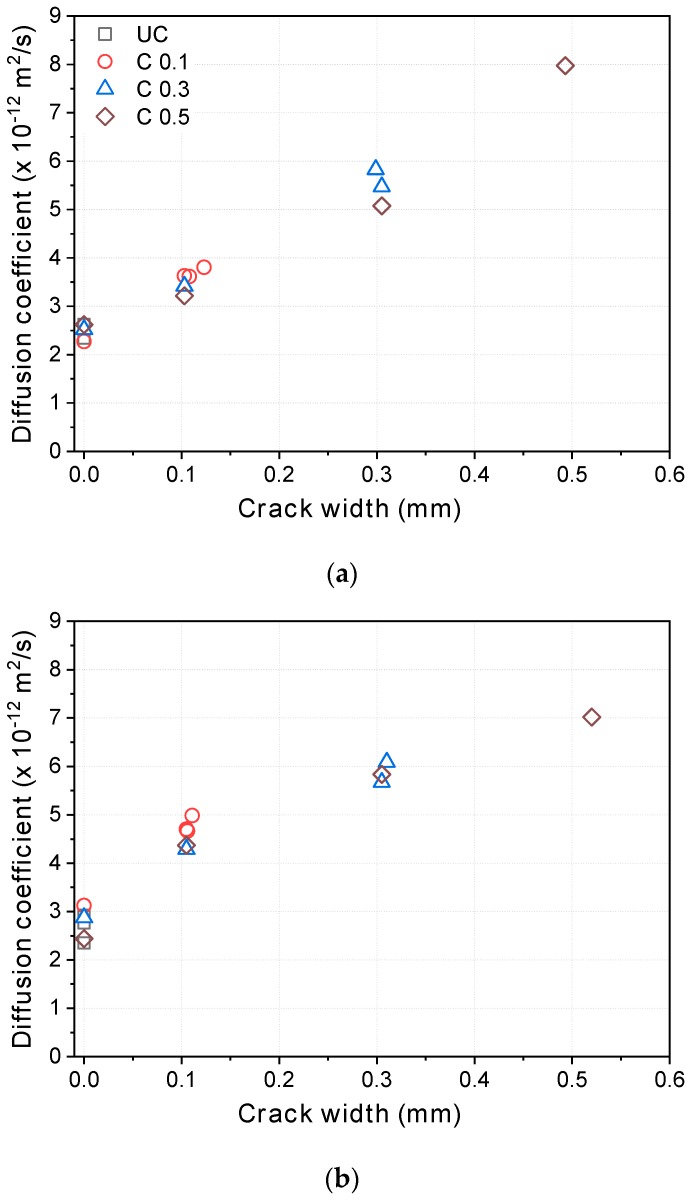

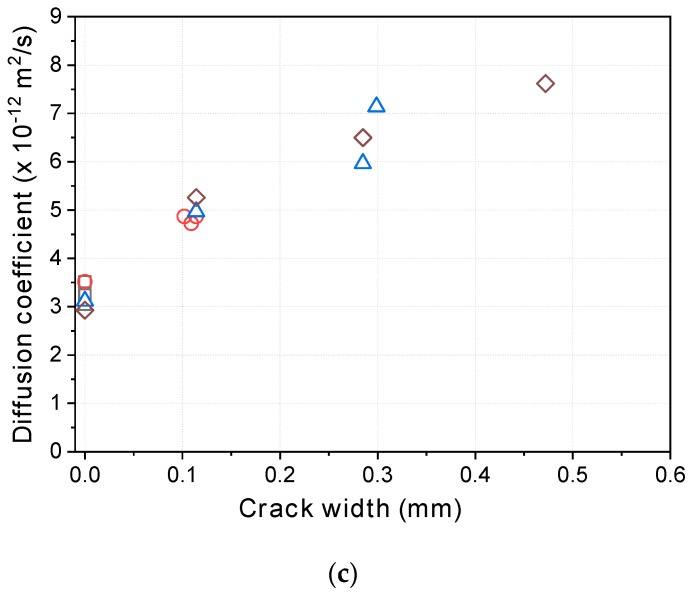
Relationship between diffusion coefficients and crack widths before healing: (**a**) OPC; (**b**) SH-A; (**c**) SH-B.

The steady-state migration diffusion coefficients can be calculated from Equation (9), given below. Figure 9 illustrates the steady-state migration diffusion coefficients according to the crack widths before healing. As expected, the diffusion coefficients increased proportionally to the crack width in an almost linear fashion. Considering the scatter of the test data from the cracked specimens, the slopes are similar, but the diffusion coefficients of the uncracked specimens are in the order of OPC < SH-A < SH-B.

From steady-state migration tests on cracked concrete, Jang et al. [1] suggested the existence of a threshold crack width at which the diffusion coefficient suddenly increased, and they attributed it to the complexity of crack geometry. However, in the present study, this was not clearly observed, possibly because of the higher voltage used here. As discussed in Section 4.1, the crack might be healed during the test under low voltage because of the relatively long test duration, and the crack healing during the test is probably more prominent for finer cracks and those with high tortuosity. As shown in Figure 6, a critical crack width does appear under the low potential (12 V).

As shown in Figure 9, the tests were repeated on the same specimens to roughly assess repeatability. The coefficients of variation (COVs) in the diffusion coefficients of the uncracked specimens are 6.2%, 8.1%, and 10.1% for OPC, SH-A, and SH-B, respectively; those in the specimens with a 0.1 mm crack are 6.3%, 9.1%, and 5.0% for OPC, SH-A, and SH-B, respectively. Thus, both sets of specimens offered good repeatability. The COVs of the diffusion coefficients of the cracked and uncracked specimens are in a similar range. The tests were also carried out on different specimens with the same mixtures. The COVs of the diffusion coefficients between the different specimens are also very low: For the uncracked specimens, 5.4%, 7.1%, and 10.0% for OPC, SH-A, and SH-B, respectively; for the specimens with a 0.1 mm crack, 6.6%, 6.9%, and 6.1% for OPC, SH-A, and SH-B, respectively.

**Figure 10:**
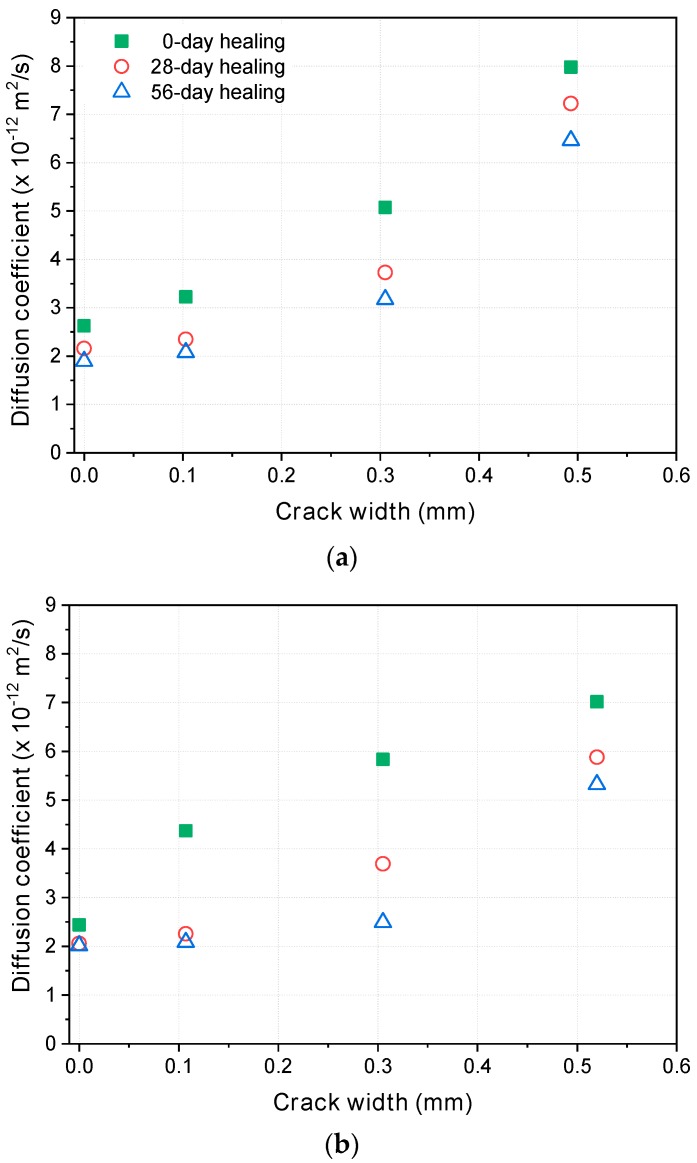

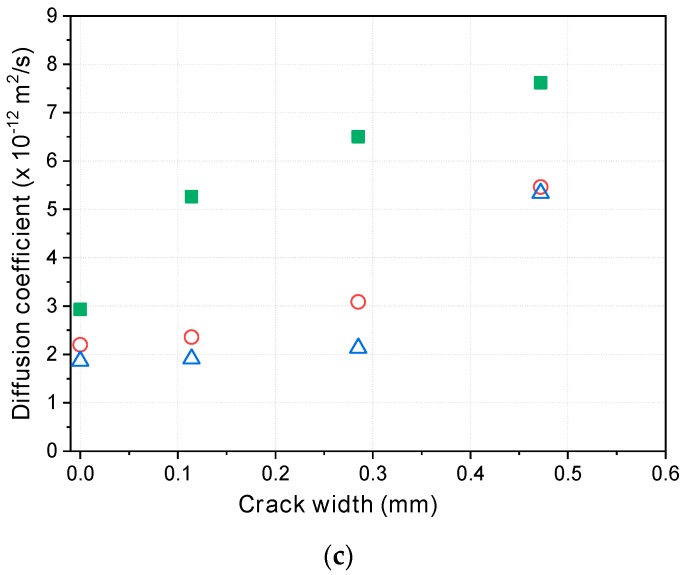
Relationship between diffusion coefficients and crack widths at different healing times: (**a**) OPC; (**b**) SH-A; (**c**) SH-B.

Figure 10 compares the calculated steady-state migration diffusion coefficients before and after healing (or additional curing for the uncracked specimens). The diffusion coefficients before healing in Figure 10 are all from different specimens, and they include some deviations, as mentioned above. Considering those deviations, it is appropriate to mention that the diffusion coefficients of the unhealed specimens are almost linearly proportional to the crack widths. After healing, however, the diffusion coefficients decreased considerably. For the specimens with a 0.1 mm crack, the diffusion coefficients after 56 days of healing were almost the same as those of the uncracked specimens, regardless of the mixture. For larger crack widths, the reduction in the diffusion coefficients depended on the mixture. For the specimens with a 0.3 mm crack, the reduction in the diffusion coefficients was in the order of OPC < SH-A < SH-B. Therefore, SH-A and SH-B have higher self-healing capacity than OPC. SH-A contains a crystalline admixture consisting of Na_2_CO_3_ and organic calcium ions impregnated in zeolite, and it is believed that they react with each other to produce CaCO_3_, which fills the space in the crack [7,50]. SH-B incorporates CSA as an expansive admixture and bentonite as a swelling agent, in addition to the crystalline admixture in SH-A. CSA is expected to create ettringite when sufficient calcium sulfate is available, and bentonite swells by absorbing water to fill the space inside a crack [7,50]. According to the present test results, the SH-B mixture has a higher self-healing capacity than the SH-A mixture. The detailed mechanisms involved in the self-healing process should be identified by a chemical analysis and an optical examination, but that was outside the scope of this study.

For the specimens with a 0.5 mm crack, the reduction in the diffusion coefficients was in the same order but lower than that of the specimens with a 0.3 mm crack, which suggests a limit in the crack width that can be healed by the self-healing techniques used in this study. 

Literature data also record the decrease of the diffusion coefficient due to self-healing similar to the results of this study [12,17,18,28,29]. Table 4 compares the steady-state diffusion coefficients from Darquennes et al. [17] and those obtained in this study. The diffusion coefficient of the OPC mixture with the water-to-cement ratio of 0.5 after seven-day curing is 2.99 × 10^−12^ m^2^/s, which is similar to the OPC data of this study. Considering higher water-to-cement ratio and shorter curing period, it seems a relatively low. This may be attributed to the longer test duration up to seven days (the potential difference was only 10 V). As shown in Table 4, the diffusion coefficient of the cracked OPC specimens with the crack width of 0.126 mm increased up to 6.43 × 10^−12^ m^2^/s, but decreased to 4.3 × 10^−12^ m^2^/s after 14 days of healing and 4.0 × 10^−12^ m^2^/s after 21 days of healing. The reduction ratio was 30.0% and 37.8%. The reduction ratio of the diffusion coefficients of the OPC mixture with the crack width of 0.106 mm of the present study was 25.1% at 28 days of healing and 35.6% at 56 days of healing, which are relatively lower than Darquennes et al.’s data. However, it is not surprising because the water-to-cement ratio was higher, and the initial curing period was only seven days. Higher unhydrated cement content could increase the self-healing capacity. The reduction ratio of the diffusion coefficients of the cracked 50% GGBFS mixture was 48.3% at 14 days of healing, and 55.3% at 21 days of healing, higher than that of the OPC mixture. This is quite similar to those of the SH-A and SH-B mixtures. However, the result of the diffusion coefficients of the cracked specimen includes the effect of the diffusion through uncracked part of the specimen. Since the diffusion coefficient of uncracked specimen also decreases with increasing age, the self-healing capacity cannot be quantitatively compared without information on the diffusion coefficient of uncracked specimen. Therefore, it is required to assess only the self-healing of crack excluding the effect of the diffusion through uncracked part.

### 4.3. Evaluation of Self-Healing Capacity

Evaluating the self-healing capacity of a specimen in a quantitative way requires a relevant index. This study proposes such an index for the recovery of resistance to chloride penetration using the diffusion coefficients determined by the steady-state chloride migration test. 

Under the steady-state condition, a cracked specimen can be represented as a two-phase parallel model divided into an uncracked part and the crack. The total ionic flow can then be given as

(5) $$Q_{tot}=Q_{ucr}+Q_{cr}$$

where $Q_{ucr}$ is the ionic flow through the uncracked part [kg/s], and $Q_{cr}$ is the ionic flow through the crack [kg/s]. Equation (5) can be rewritten as

(6) $$J_{tot}A_{tot}=J_{ucr}A_{ucr}+J_{cr}A_{cr}$$

where J is the ionic flux, i.e., the quantity of ions that crosses a certain area per unit time, and A is the area. The subscripts “*tot*”, “*ucr*”, and “*cr*” mean “total”, “uncracked”, and “crack”, respectively.

Then, because J=D ∂c/∂x,

(7) $$D_{eq}\frac{\partial c}{\partial x}A_{tot}=\;D_{ucr}\frac{\partial c}{\partial x}A_{ucr}+D_{cr}\frac{\partial c}{\partial x}A_{cr}$$

where $D_{eq}$ is the equivalent of the diffusion coefficient of chloride in the cracked mortar [m^2^/s]; $A_{tot}$ is the total surface area, including the crack, perpendicular to the direction of flow [m^2^]; $D_{ucr}$ and $D_{cr}$ are the diffusion coefficients of chloride in the uncracked zone and the crack [m^3^/s], respectively; and $A_{ucr}$ and $A_{cr}$ are the surface area of the uncracked zone and the crack perpendicular to the direction of flow [m^2^], respectively. Because the crack area is small, it can be assumed that $A_{tot}\approx A_{ucr}$, making Equation (8)

(8) $$D_{cr}A_{cr}=\left(D_{eq}-D_{ucr}\right)A_{tot}.$$

The index for the self-healing capacity in terms of resistance to chloride penetration can then be defined as

(9) $$SH_{c}\left(t\right)=1-\frac{Q_{cr}\left(t\right)}{Q_{cr,i}}=1-\frac{D_{cr}\left(t\right)A_{cr}(t)}{D_{cr,i}A_{cr,i}}=1-\frac{\left[D_{eq}\left(t\right)-D_{ucr}\left(t\right)\right]\;A_{tot}}{\left[D_{eq,i}-D_{ucr,i}\right]\;A_{tot}}\;∴\;\;\;\;SH_{c}\left(t\right)=1-\frac{D_{eq}\left(t\right)-D_{ucr}\left(t\right)}{\;D_{eq,i}-D_{ucr,i}}$$

where $Q_{cr}\left(t\right)$ and $Q_{cr,i}$ are the ionic flow at healing age t and the initial stage, respectively [kg/s]; $D_{eq}\left(t\right)$, $D_{cr}\left(t\right)$, and $D_{ucr}\left(t\right)$ are the equivalent migration coefficient of cracked mortar, the diffusion coefficient through the crack, and the diffusion coefficient of uncracked mortar at healing age t [m^2^/s], respectively; $D_{eq,i}$, $D_{ucr,i}$, and $D_{ucr,i}$ are the diffusion coefficients at the initial stage [m^2^/s]; the total area $A_{tot}$ is constant; and $A_{cr}\left(t\right)$ and *A_cr,i_* are the area of the crack perpendicular to the exposure surface at healing age and the initial stage, respectively.

**Figure 11:**
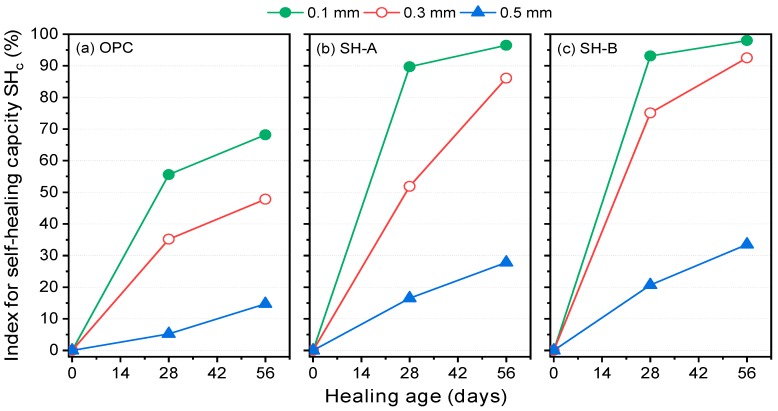
Indices for the self-healing capacity of different mixtures: (**a**) OPC; (**b**) SH-A; (**c**) SH-B.

From the diffusion coefficients given in Figure 10, the indices for self-healing capacity were determined at 28 and 56 days, as presented in Figure 11. The indices for the self-healing capacity of the OPC specimens after 56 days are 68.2%, 47.78%, and 14.67% for 0.1, 0.3, and 0.5 mm cracks, respectively. Those for the SH-A specimens are 96.5%, 86.1%, and 27.8% for 0.1, 0.3, and 0.5 mm cracks, respectively. Those for the SH-B specimens are 98.0%, 92.4%, and 33.5% for 0.1, 0.3, and 0.5 mm cracks, respectively. Clearly, using the proposed index for self-healing capacity, it is quite possible to quantitatively compare the self-healing capacities of different self-healing materials. The proposed evaluation method in the present study is compared with several existing methods in Table 5.

## 5. Conclusions

In this study, the rapid chloride ion migration test under the steady-state regime was used to assess the self-healing capacities of cracked mortar containing crystalline and expansive admixtures with respect to their resistance to chloride penetration. The following conclusions can be drawn from this study.

(1) In the steady-state migration test, the time to reach the quasi-steady-state decreased and the diffusion coefficients increased with increasing potential. This is attributed to both the effect of the potential drop inside the migration cell and the self-healing that occurred during the test. Therefore, it is necessary to minimize the test duration by choosing a high potential, though it remains important that the temperature does not rise too high during the test. Within the scope of this study, 36 V was selected as the most appropriate potential, under which the temperature rise was less than 10 °C, and the test duration could be reduced to as low as 24 to 36 h. Under those conditions, the repeatability of the test was quite high, and the deviations between different test specimens were low enough.
(2) The diffusion coefficients obtained from the steady-state diffusion coefficients under the high potential (36 V) increased almost linearly in proportion to the crack width, and the threshold crack width suggested by previous research—the point at which the diffusion coefficient suddenly increases—was not observed before healing. After a certain period of healing, the diffusion coefficients decreased regardless of the mixture type. Up to a certain crack width, the diffusion coefficients decreased to almost the same level as the uncracked specimens. This strongly suggests that the threshold crack width can be attributed to the self-healing of cracks during the test.
(3) For larger crack widths, on the other hand, the recovery of the diffusion coefficients depended on the mixture. The mixture incorporating calcium sulfoaluminate as an expansive admixture and bentonite as a swelling agent together with a crystalline admixture including Na_2_CO_3_ and organic calcium ions showed better self-healing performance than the mixture incorporating only the crystalline admixture. Also, depending on the types of self-healing techniques, it is noted that there is a limit in the crack width that can be healed.
(4) The self-healing capacity with respect to resistance to chloride penetration can be evaluated using the steady-state migration test, and an index for the recovery of resistance to chloride penetration due to self-healing can be defined using the rate of chloride ions that pass through the crack. The proposed index can be used to quantitatively compare self-healing techniques. Also, the proposed evaluation method using the steady-state migration test under higher potential can give a more accurate result because the diffusion coefficient can be measured in a relatively short time by preventing the occurrence of self-healing during the test.

## Figures and Tables

**Table 1:** Details of target crack widths in the second-phase tests.

Test Time	No. of Test Cycles	Target Crack Widths			
		“Mix. ID”-UC	“Mix. ID”-C0.1	“Mix. ID”-C0.3	“Mix. ID”-C0.5
Before healing	#1	Uncracked	Uncracked	Uncracked	Uncracked
	#2	Uncracked	0.1 mm	0.1 mm	0.1 mm
	#3	Uncracked	0.1 mm	0.3 mm	0.3 mm
	#4	Uncracked	0.1 mm	0.3 mm	0.5 mm
After healing of 28 and 56 days	#1	Uncracked	0.1 mm	0.3 mm	0.5 mm

**Table 2:** Mixture proportions, slump flow, and compressive strengths of the test specimens.

Mixture ID	Mixture Proportions (kg/m^3^)					28-Day Compressive Strength (MPa)	Slump Flow (mm)
	Water	Cement	Crystalline Admixture	Expansive Admixture	Sand		
OPC	273.2	683	-	-	1366	54.6	170
SH-A	273.2	683	20.5	-	1345.5	47.3	165
SH-B	273.2	683	20.5	20.5	1325	49.6	169

**Table 3:** Target and measured crack widths.

Potential Applied	Target Crack Width (µm)	Measured (Mean) Crack Width (µm)	Difference (%)
36 V	100	110	+10.0
	300	300	0.0
24 V	100	107	+7.0
	300	293	−2.3
12 V	100	106	+6.0
	300	312	+4.0

**Table 4:** Comparison of the diffusion coefficients with literature data.

Literature	Specimen	Mixture	Initial Curing (Days)	Diffusion Coefficient of Uncracked Specimens after Initial Curing (×10^−12^ m^2^/s)	Crack Width (mm)	Healing Age (Days)	Diffusion Coefficient of Cracked Specimens (×10^−12^ m^2^/s)	Reduction Ratio (%)
Darquennes et al. (2016) [17]	Mortar cylinder with 110 mm diameter and 30 mm thickness	100% OPC (water/cement = 0.50)	7	2.99	0.126	0	6.43	-
						14	4.50	30.0
						21	4.00	37.8
		50% OPC + 50% GGBFS (water/binder = 0.52)	7	2.90	0.152	0	7.39	-
						14	4.00	45.9
						21	3.30	55.3
This study	Mortar cylinder with 100 mm diameter and 50 mm thickness	OPC	28	2.63	0.103	0	3.23	-
						28	2.42	25.1
						56	2.08	35.6
		SH-A	28	2.44	0.114	0	4.37	-
						28	2.26	48.3
						56	2.09	52.2
		SH-B	28	2.93	0.123	0	5.26	-
						28	2.36	55.1
						56	1.91	63.7

**Table 5:** Comparison of evaluation methods of chloride penetration resistance recovery due to self-healing.

Evaluation Methods	Test Duration	Pros	Cons
Ponding test (AASHTO T259 [19], ASTM C1543 [20])	90–180 days	Natural mechanism of chloride penetration is involved.	Test duration is too long and not appropriate to evaluate the self-healing capacity. Dust sampling, chloride extraction and chemical analysis are required.
Coulomb test (ASTM C1202 [25])	6 h	Short test duration	Cannot give information about chloride diffusion.
Electrical impedance test [36,52]	5–30 min (after drying for 24 h)	Very short test duration Easy and convenient	The result strongly depends on the saturation degree of specimen To evaluate the diffusion coefficient, it is required to know the electrical conductivity of pore solution.
Non-steady-state migration test (NT Build 492 [27])	24–96 h	Relatively short test duration Easy to calculate the diffusion coefficient No need to measure the chloride concentration	Difficult to evaluate the diffusion coefficient when applied to cracked concrete because two-dimensional flow occurs and it is required to consider the chloride binding during the test.
Steady-state migration test (NT Build 355 [26])	7 days	Easy to calculate the diffusion coefficient No need to measure the chloride binding	Test duration becomes relatively long under low potential since the test should be carried out until the steady-state is reached.
The proposed method (Modified steady-state migration test)	24–36 h (under 36 V)	Relatively short test duration No need to measure the chloride binding Index for self-healing capacity can be evaluated.	To evaluate the index for self-healing capacity, the diffusion coefficients of uncracked specimens should be known.
